# The rationale, design, and methods of a randomized, controlled trial to evaluate the efficacy and safety of an active strategy for the diagnosis and treatment of acute pulmonary embolism during exacerbations of chronic obstructive pulmonary disease

**DOI:** 10.1002/clc.23161

**Published:** 2019-02-25

**Authors:** David Jiménez, Alvar Agustí, Manuel Monreal, Remedios Otero, Menno V. Huisman, José L. Lobo, Andrés Quezada, Luis Jara‐Palomares, Ascensión Hernando, Eva Tabernero, Pedro Marcos, Pedro Ruiz‐Artacho, Aitor Ballaz, Laurent Bertoletti, Francis Couturaud, Roger Yusen, on behalf of the SLICE investigators, David Jimenez, Alvar Agustí, Laurent Bertoletti, Francis Couturaud, Menno V. Huisman, David Jimenez, Jose Luis Lobo, Manuel Monreal, Remedios Otero, Roger D. Yusen, Alfonso Muriel, Ina Guerassimova, Raquel Morillo, Deisy Barrios, Andrés Quezada, Ignacio Gallego, Agustina Vicente

**Affiliations:** ^1^ Respiratory Department, Hospital Ramón y Cajal and Medicine Department Universidad de Alcalá (IRYCIS) Madrid Spain; ^2^ CIBER de Enfermedades Respiratorias (CIBERES) Barcelona Spain; ^3^ Respiratory Institute, Hospital Clinic, IDIBAPS Universitat de Barcelona Barcelona Spain; ^4^ Department of Internal Medicine, Hospital Universitari Germans Trias i Pujol, Badalona, Barcelona Universidad Católica de Murcia Murcia Spain; ^5^ Respiratory Department, Instituto de Biomedicina de Sevilla (IBiS), Centro de Investigación Biomédica en Red de Enfermedades Respiratorias (CIBERES) Hospital Universitario Virgen del Rocio Seville Spain; ^6^ Department of Thrombosis and Hemostasis Leiden University Medical Center Leiden the Netherlands; ^7^ Respiratory Department Hospital de Araba Vitoria Spain; ^8^ Respiratory Department Hospital Universitario Doce de Octubre Madrid Spain; ^9^ Respiratory Department Hospital Universitario Cruces Bilbao Spain; ^10^ Respiratory Department Hospital Universitario A Coruña A Coruña Spain; ^11^ Emergency Department Hospital Universitario Clínico San Carlos Madrid Spain; ^12^ Respiratory Department Hospital Galdakao Bilbao Spain; ^13^ Thrombosis Research Group Université de Saint‐Etienne, Jean Monnet, Inserm, CIE3. Service de Médecine Interne et Thérapeutique, Hôpital Nord, CHU de Saint‐Etienne Saint‐Etienne France; ^14^ Department of Internal Medicine and Chest Diseases, (G.E.T.B.O.), CIC INSERM University Hospital of Brest, European University of Occidental Brittany Brest France; ^15^ Divisions of Pulmonary and Critical Care Medicine and General Medical Sciences Washington University School of Medicine St. Louis Missouri; ^16^ Hospital Ramón y Cajal, IRYCIS Madrid Spain; ^17^ Hospital Clinic Barcelona Spain; ^18^ Hôpital Nord Saint‐Etienne France; ^19^ University Hospital of Brest Brest France; ^20^ Leiden University Medical Center Leiden The Netherlands; ^21^ Hospital Ramón y Cajal, IRYCIS Madrid Spain; ^22^ Hospital de Araba Vitoria Spain; ^23^ Hospital Germans Trias i Pujol Badalona Spain; ^24^ Hospital Virgen del Rocio Sevilla Spain; ^25^ University School of Medicine St. Louis Missouri USA; ^26^ Hospital Ramón y Cajal, IRYCIS Madrid Spain; ^27^ Hospital Ramón y Cajal, IRYCIS Madrid Spain; ^28^ Hospital Ramón y Cajal, IRYCIS Madrid Spain; ^29^ Hospital Ramón y Cajal, IRYCIS Madrid Spain; ^30^ Hospital Ramón y Cajal, IRYCIS Madrid Spain; ^31^ Hospital Ramón y Cajal, IRYCIS Madrid Spain; ^32^ Hospital Ramón y Cajal, IRYCIS Madrid Spain

**Keywords:** chronic obstructive pulmonary disease, exacerbation, pulmonary embolism, treatment

## Abstract

**Introduction:**

Some previous studies have suggested a high prevalence of pulmonary embolism (PE) during exacerbations of chronic obstructive pulmonary disease (ECOPD). The SLICE trial aims to assess the efficacy and safety of an active strategy for the diagnosis and treatment of PE (vs usual care) in patients hospitalized because of ECOPD.

**Methods:**

SLICE is a phase III, prospective, international, multicenter, randomized, open‐label, and parallel‐group trial. A total of 746 patients hospitalized because of ECOPD will be randomized in a 1:1 fashion to receive either an active strategy for the diagnosis and anticoagulant treatment of PE or usual care (ie, standard care without any diagnostic test for diagnosing PE). The primary outcome is a composite of all‐cause death, non‐fatal (recurrent) venous thromboembolism (VTE), or readmission for ECOPD within 90 days after enrollment. Secondary outcomes are (a) death from any cause within 90 days after enrollment, (b) non‐fatal (recurrent) VTE within 90 days after enrollment, (c) readmission within 90 days after enrollment, and (d) length of hospital stay.

**Results:**

Enrollment started in September 2014 and is expected to proceed until 2020. Median age of the first 443 patients was 71 years (interquartile range, 64‐78), and 26% were female.

**Conclusions:**

This multicenter trial will determine the value of detecting PEs in patients with ECOPD. This has implications for COPD patient morbidity and mortality.

**Trial registration number:** NCT02238639.

## 1 BACKGROUND

Chronic obstructive pulmonary disease (COPD) is a leading cause of morbidity and mortality worldwide.^1^, ^2^, ^3^ COPD patients may suffer episodes of exacerbation of symptoms (ECOPD) that contribute to poor health status, and increased healthcare costs.^4^ The majority of ECOPD cases develop in response to infections^5^, ^6^ and air pollution,^7^ but the exact cause is not clear in up to 30% of cases.^8^ In addition, other frequent clinical conditions may mimic the symptoms of ECOPD, including congestive heart failure, pneumonia, pneumothorax, pleural effusion, and pulmonary embolism (PE).^8^

Previous studies suggest a high prevalence of PE in ECOPD.^9^, ^10^, ^11^, ^12^ Tillie‐Leblond et al evaluated PE in a series of 197 consecutive patients with ECOPD and found that the frequency of PE was 25%.^13^ However, that study was performed in a highly selected subgroup of patients. In fact, a recent meta‐analysis found a lower prevalence of PE of 16% in ECOPD compared with previous studies.^14^

In patients with clinical suspicion of PE, there are some data suggesting that some PE diagnoses are less severe and these patients might not benefit from anticoagulation therapy.^15^ Particularly for patients with ECOPD, some PE might be clinically unimportant, and the risk of submitting a patient with a clinically insignificant PE to anticoagulant treatment might outweigh the benefit.^16^ Therefore, we designed the significance of puLmonary embolism in COPD exacerbations (SLICE) trial to assess the efficacy and safety of an active strategy for the diagnosis and treatment of PE compared to usual care (ie, standard care without any diagnostic test for diagnosing PE) in patients hospitalized because of ECOPD.

## 2 METHODS

SLICE complies with the standard protocol items: recommendations for interventional trials statement.^17^

### 2.1 Study hypothesis

This trial is designed to demonstrate the superiority of an active strategy for the diagnosis and treatment of PE compared to usual care in patients hospitalized because of ECOPD.

### 2.2 Trial design and patient population

**Figure 1:**
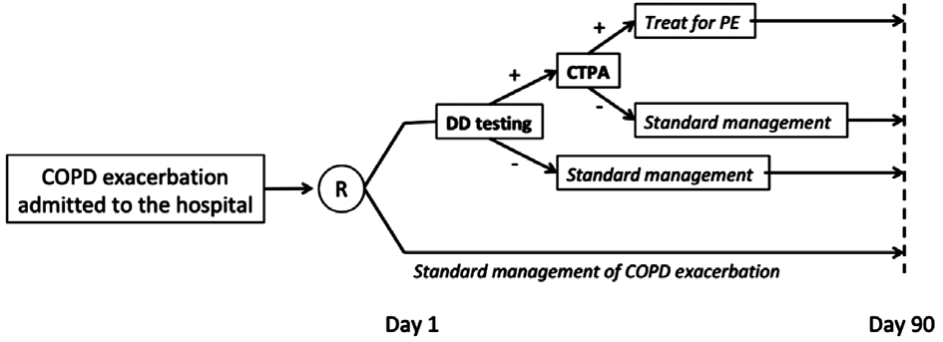
Study flowchart

SLICE is an investigator‐initiated, phase III, prospective, international, multicenter, randomized (1:1), open‐label with blind end‐point evaluation (PROBE), parallel‐group trial (ClinicalTrials.gov identifier NCT02238639). Consecutive adult patients with ECOPD who require hospital admission are eligible for the study. The inclusion and exclusion criteria of the SLICE trial are listed in Table 1; the flow diagram is displayed in Figure 1. The study information for all ineligible and eligible non‐recruited participants will be retained in an anonymized form to provide detailed data on these patients in comparison to the study participant population. The study is being conducted in 16 centers in Spain and France.

**Table 1:** Eligibility criteria

Inclusion criteria
Previous diagnosis of COPD: post‐bronchodilator forced expiratory volume in 1 second (FEV1)/forced vital capacity (FVC) < 0.7
Hospital admission because COPD exacerbation without initial clinical suspicion of PE in the Emergency Department (according to the Emergency Department physician evaluation)
Exclusion criteria
Unable to provide informed consent
Contraindication to a contrast‐enhanced, PE‐protocol, multidetector computerized tomography (CTPA): allergy to intravenous contrast medium, or renal failure defined as a creatinine clearance <30 mL/min, based on the Cockroft‐Gault equation
Anticoagulant therapy at the time of hospital admission
Pregnancy, or breast feeding
Life expectancy of less than 3 months
Diagnosis of pneumothorax, or pneumonia (fever [temperature ≥ 38°C], and purulent sputum, and new infiltrate in chest X‐ray)
Diagnosis of lower respiratory tract infection (fever [temperature ≥ 38°C], increased sputum volume and/or increased sputum purulence)
Indication of invasive mechanical ventilation at the time of hospital admission
Inability to comply with study assessments

### 2.3 Randomization and trial interventions

In a patient with ECOPD who requires hospital admission, randomization should occur in the first 24 hours after admission. The trial uses a computer‐generated randomization scheme. Randomization is stratified by center and, within the centers, performed in blocks of 4 and 6 to ensure balanced distribution of the management groups. Randomization is performed centrally through the Internet (www.estudioslice.org), and management allocation is concealed from all investigators.

#### 2.3.1 Intervention group

Patients in the intervention group have blood samples collected from an antecubital vein, and undergo D‐dimer testing within 12 hours after randomization. Cutoff levels for defining elevated D‐dimer are defined by the Department of Clinical Chemistry at each participating site. For patients with a negative D‐dimer, a diagnosis of PE is ruled out. For patients with a positive D‐dimer, a contrast‐enhanced, PE‐protocol, multidetector computerized tomography (CTPA) is performed. CTPA results are categorized as positive for PE if an intraluminal filling defect is seen in (sub)segmental or more proximal branches, and are considered negative if no filling defect is observed. Scans are considered technically inadequate only if main or lobar pulmonary vessels are not visualized. Although not mandatory, the protocol suggests the use of complete lower limb compression ultrasonography (CCUS) to detect concomitant deep vein thrombosis (DVT) for patients with isolated subsegmental PE.

If the diagnosis of PE is confirmed, patients receive anticoagulant treatment according to guideline recommendations: parenteral anticoagulation (ie, unfractionated heparin, low‐molecular‐weight heparin, or fondaparinux) overlapped and followed by vitamin K antagonists; or parenteral anticoagulation followed by dabigatran or edoxaban; or monotherapy with apixaban or rivaroxaban.^18^

#### 2.3.2 Control group

Patients in the control group undergo standard (ie, according to clinical practice guidelines) clinical management,^1^, ^8^ as deemed appropriate by the attending physician.

### 2.4 Study outcomes

The primary efficacy outcome is the composite of death from any cause, non‐fatal (recurrent) symptomatic venous thromboembolism (VTE), or readmission for ECOPD within 90 days after enrollment. Confirmation of (recurrent) symptomatic PE requires symptoms of PE and a new or an extension of a previous intraluminal‐filling defect in (sub)segmental or more proximal branches on PE‐protocol chest CTPA. Confirmation of (recurrent) symptomatic DVT requires symptoms of DVT and the following criteria: (a) In the absence of previous DVT investigations at baseline, a non‐compressible venous segment on ultrasonography, (b) if there were previous DVT investigations at baseline, abnormal lower limb CCUS where compression had been normal; or, if previously non‐compressible, a substantial increase (≥4 mm) in diameter of the thrombus during full compression.

Secondary efficacy outcomes include: (a) death from any cause within 90 days after enrollment, (b) non‐fatal (recurrent) symptomatic VTE within 90 days after enrollment, (c) readmission for ECOPD within 90 days after enrollment, and (d) length of hospital stay.

The principal safety outcome is major bleeding within 90 days after enrollment, defined according to the guidelines of the International Society of Thrombosis and Hemostasis,^19^ as acute clinically overt bleeding associated with one or more among the following: a decrease in hemoglobin of 2 g/dL or more, a transfusion of two or more units of packed red blood cells, bleeding that occurs in at least one of the following critical sites (intracranial, intraspinal, intraocular, pericardial, intraarticular, intramuscular with compartment syndrome or retroperitoneal), bleeding that is fatal (defined as a bleeding event that the central independent committee adjudicate as the primary cause of death or contributing directly to death) and bleeding that necessitates surgical intervention. A bleeding event is classified as a clinically relevant non‐major bleeding event if it is overt (ie, is symptomatic or visualized by examination) not meeting the criteria for major bleeding, requires medical attention or is associated with discomfort for the subject such as pain, or impairment of activities of daily life.

A central independent adjudication committee whose members are unaware of management allocation adjudicates all suspected study outcomes during the study period.

### 2.5 Surveillance and follow‐up

The study requires the following scheduled visits: enrollment, 1 week, 1 month, and 3 months after randomization. Additional visits are performed if new symptoms and/or signs of VTE or major bleeding occur during the study period or anytime it is deemed necessary by the investigator. Clinical examination, laboratory and diagnostic imaging are performed if the patient develops symptoms or signs suggestive of (recurrent) VTE.

### 2.6 Sample size of the study

Previous studies have shown short‐term rates of death, thromboembolic events, or readmission of approximately 40% at day 90 among patients who required hospital admission because of ECOPD.^20^ An estimated 355 participants will be needed in each trial group to detect a clinically important 10% absolute reduction in the primary outcome (ie, from 40% to 30%) with 80% power at 5% significance level. The 10% reduction was based on consultation with primary and secondary care colleagues (general practitioners and pulmonologists) who considered a 10% reduction to be small but clinically important. Since an interim analysis showed that 3% of patients were lost to follow‐up, the Steering Committee anticipated a 5% loss to follow‐up. This inflated each study group to 373 patients, giving 746 patients in total.

### 2.7 Statistical analysis

All analyses will be performed on the intention‐to‐treat population, defined as all patients randomized, regardless of the management actually received. A per‐protocol analysis, excluding protocol violations, will be performed as a sensitivity analysis. The distribution of continuous variables will be assessed by the Kolmogorov‐Smirnov test. Categorical variables are expressed as frequencies or percentages and compared by χ^2^ statistics or Fisher's exact test. Continuous variables will be summarized as the means ± SD or median and compared using Student's *t* test (for normal data) and Mann‐Whitney *U* test (for non‐normally distributed variables). Survival curves with time‐to‐event data will be generated by the Kaplan‐Meier method and compared using the log‐rank test. Comparisons between the two groups will be performed using the Cox proportional hazard model. A *P*‐value <0.05 will be considered statistically significant. All analyses will be performed with the use of the statistical programme SPSS V.24.0.

Subgroup analyses will include: age (<75 vs ≥75 years), sex (female vs male), COPD severity (FEV1 > 80%, 50% < FEV1 < 80%, 30% < FEV1 < 50%, and FEV1 < 30%), hospital volume (<300 beds vs ≥300 beds), and season of the year (autumn, winter, spring, and summer).

Two sensitivity analyses are planned for the primary outcome. The first is an analysis of primary‐outcome events after excluding those patients in the intervention group with a diagnosis of isolated sub‐segmental PE. The second is an analysis of outcomes after excluding patients with a history of cancer.

### 2.8 Study organization

The SLICE is an independent, investigator‐initiated trial with an academic sponsor (Respiratory Department, Ramon y Cajal Hospital). The Steering Committee (listed in the Appendix) assesses the progress, provide scientific input, and address policy issues and operational aspects of the protocol and recommendations of the Data and Safety Monitoring Board (DSMB). At the end of the trial, the Steering Committee will meet in a closed session to discuss the trial results. Data are collected, maintained and will be analyzed by S&H Medical in Spain under the supervision of the Steering Committee members.

### 2.9 Study Committees

The structure of the SLICE study includes a Steering Committee, a central independent adjudication committee, and a DSMB.

The Steering Committee members have the final responsibility for the conduction of the study as well as the verification and analyses of all the study data. All the members of the Steering Committee have access to the study data, vouch for their accuracy, and completeness; they will contribute to the interpretation of the results, approve the final version of the manuscript verifying the fidelity of the article to the study protocol, and make the decision to submit the manuscript for publication.

### 2.10 Adjudication committee

A central independent adjudication committee, whose members are unaware of management allocation, adjudicates all suspected outcome events (see Outcomes).

### 2.11 Data and safety monitoring board

An independent DSMB periodically reviews the study outcomes with all information available concerning management allocation. The DSMB is composed of three expert clinicians with experience in the conduction and monitoring of clinical trials.

### 2.12 Ethics and dissemination

The study is performed in accordance with the provisions of the Declaration of Helsinki and local regulations. Protocol and amendments have to be approved by the Institutional Review Board or Ethic Committee at each study center. The protocol and informed consent have been approved by the Institutional Review Board of Ramon y Cajal Hospital, and accepted by each participating center. Written informed consent for participation in the trial is obtained from all enrolled patients. Dissemination of the results will include conference presentations and publications in peer‐reviewed journals.

## 3 RESULTS

Enrollment started in September 2014 and is expected to proceed until 2020. Median age of the first 443 enrolled patients was 71 years (interquartile range, 64‐78), and 26% of patients were female.

## 4 DISCUSSION

COPD patients may suffer from exacerbations, defined by an acute worsening of respiratory symptoms beyond normal day‐to‐day variations and leading to a change in medication.^8^ Exacerbations are frequent (about one in four patients experience at least 2 exacerbations per year^21^), and are major determinants of health status in COPD. COPD exacerbations requiring hospital admission are independent predictors of mortality in COPD^22^ and also drive disease progression, with approximately 25% of the lung function decline attributed to exacerbations.^23^

The SLICE trial is currently enrolling patients to assess the efficacy and safety of an active strategy for the diagnosis and treatment of PE in patients with ECOPD. The trial has the potential to improve the management of exacerbations in patients with COPD. It is anticipated that the findings of this study will enhance our understanding of the exacerbations of COPD. This rigorously designed trial will address the role of PE in the decompensation of patients with COPD, potentially leading to better care.

Previous studies and meta‐analyses have assessed the prevalence of PE in ECOPD.^9^, ^10^, ^11^, ^12^, ^13^, ^14^ However, it is not known if all these PEs are clinically important. The broad use of CTPA for the diagnosis of PE has had minimal impact on the overall mortality related to PE, suggesting that some extra cases of PE may not have been clinically relevant.^24^ To the best of our knowledge, this is the first randomized controlled trial that will determine the value of detecting PEs in patients with ECOPD.^25^

Our trial has some limitations. This is an open‐label trial, and ascertainment bias is inherent to the trial design. To mitigate potential bias, all events are adjudicated by a committee whose members are unaware of the intervention assignments. The decision to use a composite outcome that includes readmissions for ECOPD might prove challenging for the interpretation of results. There are some reasons for including readmission as an outcome in the study protocol. First, exacerbations of COPD are associated with accelerated loss of lung function and death.^26^ Second, management of these outcomes may reduce the risk of reaching other endpoints (mainly death). Finally, some of readmissions for ECOPD might be caused by thromboembolic events. Thus, the Steering Committee felt justified in using a composite outcome that includes (recurrent) VTE and readmission for ECOPD. In addition, the components of the composite variable will be also analyzed separately.

In conclusion, the SLICE trial will provide high‐quality evidence regarding the risks as well as the benefits of using CTPA in the evaluation of ECOPD.

## CONFLICTS OF INTEREST

The authors declare no potential conflict of interests.

## Supporting information


**APPENDIX S1**

## Appendix

**Coordinator of the SLICE trial:** David Jimenez (Hospital Ramón y Cajal, IRYCIS, Madrid, Spain).

**SLICE Steering Committee Members**: Alvar Agustí (Hospital Clinic, Barcelona, Spain), Laurent Bertoletti (Hôpital Nord, Saint‐Etienne, France), Francis Couturaud (University Hospital of Brest, Brest, France), Menno V. Huisman (Leiden University Medical Center, Leiden, The Netherlands), David Jimenez (Hospital Ramón y Cajal, IRYCIS, Madrid, Spain), Jose Luis Lobo (Hospital de Araba, Vitoria, Spain), Manuel Monreal (Hospital Germans Trias i Pujol, Badalona, Spain), Remedios Otero (Hospital Virgen del Rocio, Sevilla, Spain), Roger D. Yusen (University School of Medicine, St. Louis, Missouri, USA).

**Data and Safety Monitoring Board:** Alfonso Muriel (Hospital Ramón y Cajal, IRYCIS, Madrid, Spain), Ina Guerassimova (Hospital Ramón y Cajal, IRYCIS, Madrid, Spain), Raquel Morillo (Hospital Ramón y Cajal, IRYCIS, Madrid, Spain).

**Adjudication Committee:** Deisy Barrios (Hospital Ramón y Cajal, IRYCIS, Madrid, Spain), Andrés Quezada (Hospital Ramón y Cajal, IRYCIS, Madrid, Spain), Ignacio Gallego (Hospital Ramón y Cajal, IRYCIS, Madrid, Spain), Agustina Vicente (Hospital Ramón y Cajal, IRYCIS, Madrid, Spain).
