# Supplementary material for: The rationale, design, and methods of a randomized, controlled trial to evaluate the efficacy and safety of an active strategy for the diagnosis and treatment of acute pulmonary embolism during exacerbations of chronic obstructive pulmonary disease
Source: Clin Cardiol. 2019 Feb 25;42(3):346–51. doi: 10.1002/clc.23161 (PMC6712316; doi:10.1002/clc.23161)
Supplement: Supplementary file 1 — APPENDIX S1 [file CLC-42-346-s001.docx]

**Supplementary Appendix**

The rationale, design, and methods of a randomized, controlled trial to evaluate the efficacy and safety of an active strategy for the diagnosis and treatment of acute pulmonary embolism during exacerbations of chronic obstructive pulmonary disease

Authors:

David Jiménez^1^, PhD, Alvar Agustí^2, 3^, PhD, Manuel Monreal^4^, PhD, Remedios Otero^5^, PhD, Menno V. Huisman^6^, MD, José Luis Lobo^7^, MD. Andrés Quezada^1^, MD, Luis Jara-Palomares^5^, PhD, Ascensión Hernando^8^, MD, Eva Tabernero^9^, MD, Pedro Marcos^10^, MD, Pedro Ruiz-Artacho^11^, PhD, Aitor Ballaz^12^, MD, Laurent Bertoletti^13^, MD, Francis Couturaud^14^, MD, Roger Yusen^15^, MD on behalf of the SLICE investigators

**Correspondence:**

David Jiménez

Respiratory Department and Medicine Department

Ramón y Cajal Hospital, IRYCIS and Alcalá de Henares University

28034 Madrid, Spain

Phone: +34913368314

e-mail: [djimenez.hrc@gmail.com](mailto:djimenez.hrc@gmail.com)

**Table of Contents**

**A. Investigators**

A1. Participating centers

**B. Methods**

B1. Inclusion criteria

B2. Exclusion criteria

B3. Outcome definitions

**A. Investigators:**

**A1. Participating centers**

Hospital Ramón y Cajal, David Jiménez

Hospital Virgen del Rocío, Luis Jara-Palomares

Hospital Cruces, Eva Tabernero

Hospital Doce de Octubre, Ascensión Hernando

Hospital Clínico San Carlos, Pedro Ruiz-Artacho

Hospital Araba, José Luis Lobo

Fundación Jiménez Díaz, María Jesús Rodríguez Nieto

Complejo Universitario A Coruña, Pedro Jorge Marcos

Hospital Galdakao, Aitor Ballaz

Hospital Marqués de Valdecilla, Ramón Agüero

Hospital Clinic de Barcelona, Sonia Jiménez

Hospital La Fe, Raquel López

Hospital La Rosaleda, Andrés Vilas

Hospital La Paz, Antonio Emilio Martínez

Université de Saint-Etienne, Laurent Bertoletti

University Hospital of Brest, Francis Couturaud

**B. Methods:**

**B1. Inclusion criteria for SLICE**

- Previous diagnosis of COPD: post-bronchodilator forced expiratory volume in one second (FEV1) / forced vital capacity (FVC) < 0.7.
- Hospital admission because COPD exacerbation without initial clinical suspicion of PE in the Emergency Department (according to the Emergency Department physician evaluation).

**B2. Exclusion criteria for SLICE**

- Unable to provide informed consent.
- Contraindication to a contrast-enhanced, PE-protocol, multidetector computerized tomography (CTPA): allergy to intravenous contrast medium, or renal failure defined as a creatinine clearance < 30 mL/min, based on the Cockroft-Gault equation.
- Anticoagulant therapy at the time of hospital admission.
- Pregnancy, or breast feeding.
- Life expectancy of less than 3 months.
- Diagnosis of pneumothorax, or pneumonia (fever [temperature > 38ºC], and purulent sputum, and new infiltrate in chest X-ray).
- Diagnosis of lower respiratory tract infection (fever [temperature > 38ºC], increased sputum volume and/or increased sputum purulence).
- Indication of invasive mechanical ventilation at the time of hospital admission.
- Inability to comply with study assessments.

**B3. Outcome definitions**

Adjudicators will use medical record review to assess vital status. For patients who die, further medical record review, and proxy interviews when necessary, will assist with determination of the date and cause of death. Pulmonary embolism will be considered to be the cause of death if there is objective documentation that pulmonary embolism caused the death or if the death cannot be attributed to a documented cause and pulmonary embolism cannot be ruled out.

Confirmation of (recurrent) symptomatic PE requires symptoms of PE and a new or an extension of a previous intraluminal-filling defect in (sub)segmental or more proximal branches on PE-protocol chest CTPA.

Confirmation of (recurrent) symptomatic deep vein thrombosis (**DVT**) requires symptoms of DVT and the following criteria: 1) In the absence of previous DVT investigations at baseline, a non-compressible venous segment on ultrasonography; 2) if there were previous DVT investigations at baseline, abnormal complete lower limb compression ultrasonography (**CCUS**) where compression had been normal; or, if previously non-compressible, a substantial increase (>4 mm) in diameter of the thrombus during full compression.

Major bleeding is defined as acute clinically overt bleeding associated with one or more among the following: a decrease in hemoglobin of 2 g/dL or more, a transfusion of two or more units of packed red blood cells, bleeding that occurs in at least one of the following critical sites (intracranial, intraspinal, intraocular, pericardial, intraarticular, intramuscular with compartment syndrome or retroperitoneal), bleeding that is fatal (defined as a bleeding event that the independent central committee adjudicate as the primary cause of death or contributing directly to death) and bleeding that necessitates surgical intervention.

A bleeding event will be classified as a clinically relevant non-major bleeding event if it is overt (i.e., is symptomatic or visualized by examination) not meeting the criteria for major bleeding, requires medical attention or is associated with discomfort for the subject such as pain, or impairment of activities of daily life.
